# Chronic Upper Airway Obstruction Induces Abnormal Sleep/Wake Dynamics in Juvenile Rats

**DOI:** 10.1371/journal.pone.0097111

**Published:** 2014-05-13

**Authors:** Gideon Gradwohl, Nilly Berdugo-Boura, Yael Segev, Ariel Tarasiuk

**Affiliations:** 1 Sleep-Wake Disorders Unit, Soroka University Medical Center and Department of Physiology, Faculty of Health Sciences, Ben-Gurion University of the Negev, Beer-Sheva, Israel; 2 Shraga Segal Department of Microbiology and Immunology, Faculty of Health Sciences, Ben-Gurion University of the Negev, Beer-Sheva, Israel; 3 Unit of Biomedical Engineering, Department of Physics, Jerusalem College of Technology, Jerusalem, Israel; Texas Christian University, United States of America

## Abstract

**Objectives:**

Conventional scoring of sleep provides little information about the process of transitioning between vigilance-states. We used the state space technique to explore whether rats with chronic upper airway obstruction (UAO) have abnormal sleep/wake states, faster movements between states, or abnormal transitions between states.

**Design:**

The tracheae of 22-day-old Sprague-Dawley rats were surgically narrowed to increase upper airway resistance with no evidence for frank obstructed apneas or hypopneas; 24-h electroencephalography of sleep/wake recordings of UAO and sham-control animals was analyzed using state space technique. This non-categorical approach allows quantitative and unbiased examination of vigilance-states and state transitions. Measurements were performed 2 weeks post-surgery at baseline and following administration of ritanserin (5-HT2 receptor antagonist) the next day to stimulate sleep.

**Measurements and Results:**

UAO rats spent less time in deep (delta-rich) slow wave sleep (SWS) and near transition zones between states. State transitions from light SWS to wake and vice versa and microarousals were more frequent and rapid in UAO rats, indicating that obstructed animals have more regions where vigilance-states are unstable. Ritanserin consolidated sleep in both groups by decreasing the number of microarousals and trajectories between wake and light SWS, and increasing deep SWS in UAO.

**Conclusions:**

State space technique enables visualization of vigilance-state transitions and velocities that were not evident by traditional scoring methods. This analysis provides new quantitative assessment of abnormal vigilance-state dynamics in UAO in the absence of frank obstructed apneas or hypopneas.

## Introduction

Sleep disordered breathing is relatively common and, if left untreated, may lead to a substantial cascade of complex endocrine derangements that affect longitudinal growth, sleep, energy metabolism, and behavior [1]–[8]. Upper airway obstruction (UAO) by tracheal narrowing in rats leads to adaptive changes in the respiratory system, including large swings in pleural pressure and respiratory muscle contractility [9]–[16]. These adaptive changes are essential for proper ventilation maintenance especially during sleep [17]–[19] (a condition where respiratory drive may not be sufficient to support loaded breath), which ultimately leads to inadequate sleep and energy metabolism abnormalities during UAO in rats [20].

However, traditional sleep scoring reveals little information about sleep-wake dynamics and respiratory effort-related sleep fragmentation during UAO in an animal model without evidence for frank apneas or hypopneas or abnormal gas exchange values [9]–[16], [20]. Traditional sleep scoring reveals little information about the process of transitioning between vigilance-states, as cortical activity and behavior can change quite rapidly in rodents. Also, conventional scoring simply identifies discrete states, so it can overlook important variations within states, such as the distinctions between light and deep slow wave sleep (DSWS), or often excludes or dilutes events through averaging. The previous application of state space techniques (SST) of sleep recordings used local field potential data, but the variability in these signals prevented comparisons between animals [21]–[23]. Recently SST was developed to enable inter-animal comparisons of electroencephalography (EEG) dynamics of sleep/wake behavior [24].

The effect of UAO in rats on EEG dynamics of vigilance-states using SST was not explored. We hypothesized that increased respiratory efforts during UAO, in the absence of frank obstructive apneas or hypopneas, will lead to sleep/wake instability. In the present study we explored the effect of UAO on sleep state stability by using the SST at baseline and following stimulation of sleep depth with ritanserin (5-HT2 receptor antagonist) [16], [25]–[27]. We used SST to explore whether the abnormal sleep in UAO rats due to respiratory efforts reflects unstable sleep, faster movements between states, or abnormal microarousal and transitions between states.

## Methods

### Animals

Male Sprague-Dawley rats 22-days-old (53–55 gr) were used. Animals were kept on 12–12 light-dark cycle with lights on 09∶00. Rats were housed individually in Plexiglas cages at 23±1.0°C. Food and water were given *ad libitum.* The study was approved by the Ben-Gurion University of the Negev Animal Use and Care Committee and complied with the American Physiological Society Guidelines.

### Surgery

The technique used for sham surgery and to induce upper airway obstruction (UAO) in 22-day-old male rats was previously described [9], [10], [13]–[16], [20]. Animals were anesthetized with tribromoethanol (200 mg/kg) administered intraperitoneally. A midline ventral cervical incision was made to the trachea, which was exposed and dissected in order not to damage adjacent structures. A circumferential silicon band 0.5 cm long was placed around the trachea to induce tracheal-narrowing. Two sutures were looped around the band and tightened, thus constricting the trachea to increase inspiratory esophageal pressure swings two–three-fold. Controls underwent sham surgery with no tracheal narrowing. On day 7 after UAO/sham surgery a telemetric transmitter (TL11M2-F20-EET Data Sciences International, DSI, St. Paul, MN, USA, bandwidth of 0.5–50 Hz) was implanted under sterile conditions, enabling recording of EEG, dorsal neck electromyography (EMG), and body temperature. Leads from the electrodes for EEG recording were placed over the frontal (1.1 mm anterior and 1.1 mm lateral to the bregma) and parietal (3 mm posterior and 1.5 mm lateral to the bregma) cortices. EEG electrodes were anchored to the skull with dental cement [16]. Following surgery, prophylactic enrofloxacin 5 mg/mL (s.c.) and water containing ibuprofen (0.1 mg/mL) were given for three days [15], [16], [20].

### Ritanserin Study

Animals were acutely administered i.p. with high dose (2 mg/kg) ritanserin (Sigma-Aldrich Ltd., Israel) or vehicle (4% methyl alcohol in saline) at lights on [15], [16].

### Experimental Schedule

On day 16 post-surgery, sleep was recorded after acute administration of vehicle and this served as a baseline; on the following day sleep was recorded after acute administration of ritanserin, a 5-HT2 receptor antagonist, at lights on. On day 18 animals were killed.

### Sleep-wake Recording

Raw EEG and EMG outputs from the skull and skeletal muscle electrodes were sampled at 250 Hz, filtered at 0.5–40 Hz and 10–300 Hz, respectively, using DSI system (DSI, St. Paul, MN, USA) [16], [20].

### Conventional Scoring of Vigilance-states

**Figure 1:**
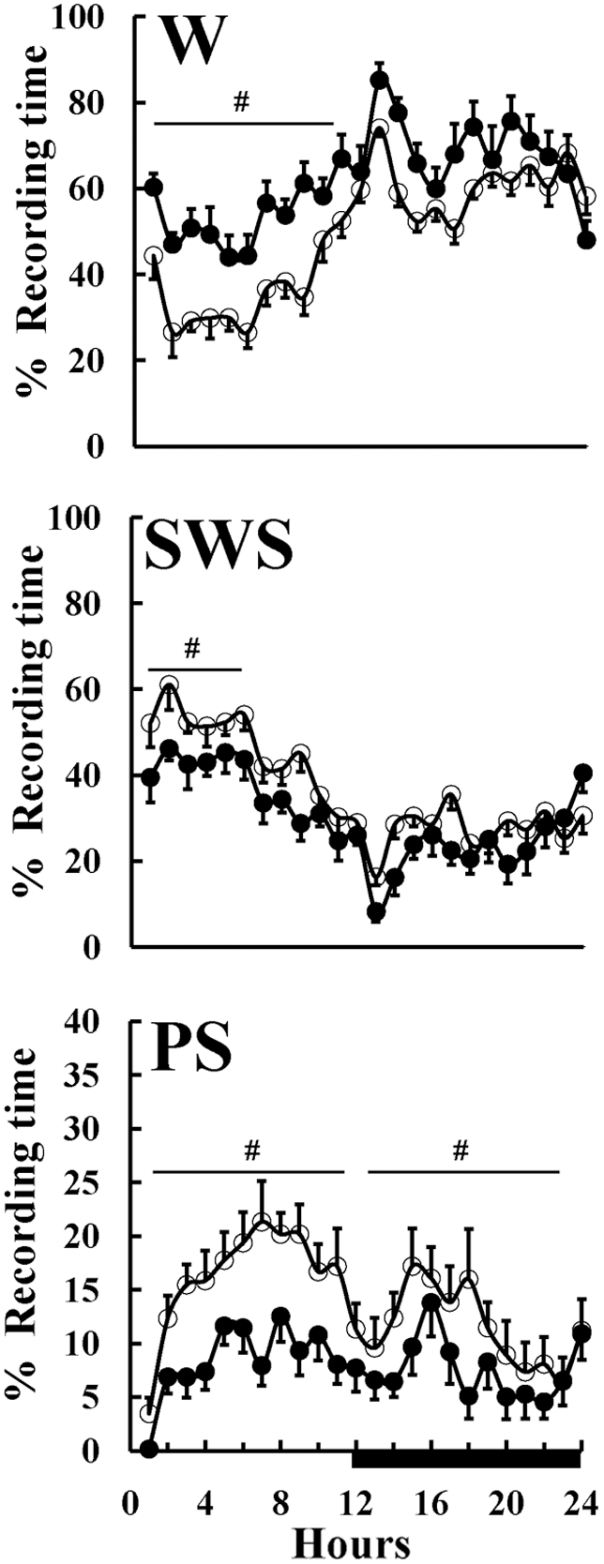
Spontaneous sleep in control and obstructive rats. Hourly values of wake (W), slow wave sleep (SWS), and paradoxical sleep (PS) are shown. Controls (n = 9) - open circles, Obstructive (n = 10) rats -filled circles. Black horizontal bars represent the light-off (active) period on a 12∶12-h cycle lights on at 09∶00. Upper airway obstruction (UAO) group had significantly more wake and less SWS and PS than controls during light period. During dark period obstructive group had significantly less PS than controls. #Indicates statistically significant (*p*<0.01) difference between the groups, ANOVA-2. Values are mean±SEM.

Vigilance-states were scored using DSI NeuroScore (v. 2.1 software) and edited visually for 10-second epochs, on the basis of the predominant state within the epoch [16], [20], [28], [29]. The duration of sleep-wake states was calculated in 1 hour time intervals (Figure 1). The sleep was categorized as: 1) Wake, 2) Slow wave sleep (SWS), and 3) Paradoxical sleep (PS). SWS was defined as high-amplitude EEG waves, lack of body movement, and predominant EEG power in the delta range (0.5–4.0 Hz); Light SWS (LSWS) was defined as high-voltage slow cortical waves (0.5–4 Hz) interrupted by low-voltage fast EEG activity (spindles, 6–15 Hz). Deep SWS (DSWS) was defined as continuous (>70% epoch) high-amplitude slow cortical waves (0.5–4 Hz) with reduced EMG and motor activity. Paradoxical sleep (PS) – highly regular low-amplitude EEG, dominance of theta activity with corresponding high fast theta (5.0–8.0 Hz) power, general lack of body movements with occasional twitches; Wake – less regular low-amplitude EEG, lack of visible theta dominance, and frequent body movements.

### Construction of the Two-dimensional State Space

**Figure 2:**
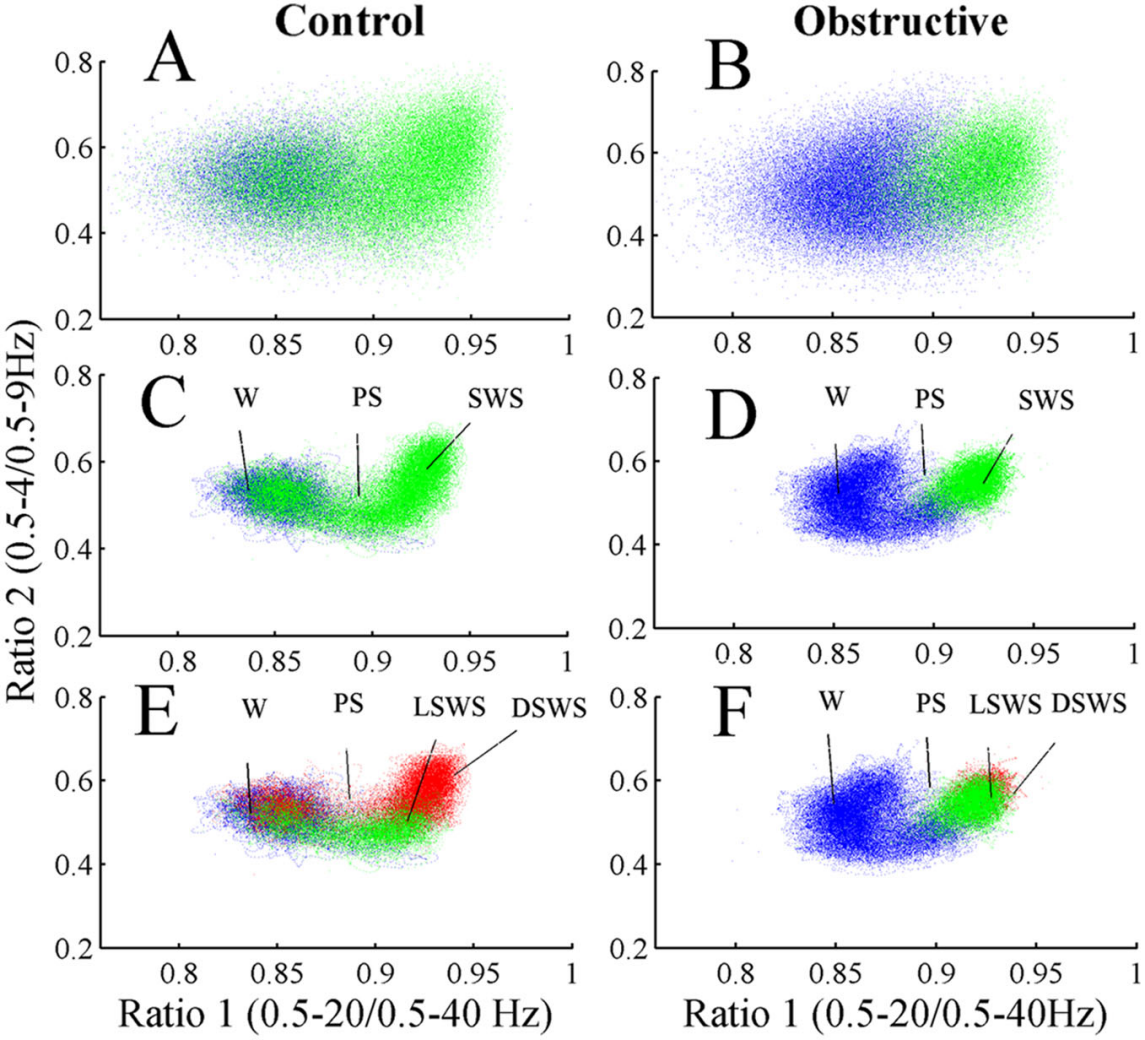
Spectral ratios of EEG activity define a 2-dimensional state space with distinct clusters. (A) control and (B) UAO; spectral ratios of EEG activity define a 2D state space with distinct clusters. Each plot shows 24 hours of EEG activity, and each point represents 1 second of EEG activity. (C) and (D) show 2D state space after application of a Hanning window (20 seconds) of one control and one UAO animal, respectively. Panels A–D: Blue–Wake; Green–slow wave sleep (SWS); Black–paradoxical sleep (PS). Panels E and F: light SWS (LSWS)–green; deep SWS (DSWS)–red.

Using an approach similar to previous reports [21], [24], we defined a 2-dimensional (2D) state space using 2 spectral amplitude ratios calculated by dividing integrated spectral amplitudes at selected frequency bands. A sliding window Fourier transform was applied to each raw (0.5–250 Hz) EEG signal using a 2-second window with a 1 second step size. Then we calculated two spectral amplitude ratios by integrating the spectral amplitude over specific frequencies: 0.5–20/0.5–40 Hz for ratio 1 (plotted on the abscissa) and 0.5–4/0.5–9 Hz for ratio 2 (plotted on the ordinate). The ratios derived from 24-hour EEG recordings were smoothed with a 20-second wide Hanning window. Each second of this smoothed ratio was mapped into the 2D state space, ratio 2 vs. ratio 1. A graphical user interface was developed to validate the 2D SST by comparing the clustering of 2D state space against the conventionally scored vigilance-states. A general agreement between these two methods was found; distinct clusters of points correspond to distinct states of wake, SWS, and PS (Figure 2). SST yields wake at low ratio 1 values and SWS at high ratio 1 values.

### State Space Densities

To calculate state space densities, the state space was binned using a grid of 1000×1000 uniformly spaced boxes, and the average density of points in each box was calculated. Therefore, the density of points reflects the relative abundance of the different behavioral states. The state space densities graph was projected to ratio 1. The densities plotted due to this projection reflect the distribution of the sum of ratio 2 vs. ratio 1.

### State Space Velocities

For each animal, we created state space velocity maps to determine rates of change across the state space. The speed of the spectral change was calculated as the distance between two consecutive data points (calculated from the horizontal and vertical velocities by the Pythagorean theorem), and in this sense, the temporal resolution was second by second. After this calculation, we mapped the velocities to a 1000×1000 grid with the values at each point representing the average velocities originating at that site.

### Trajectory Analysis

To understand the patterns of EEG activity as rats move between stable states, we identified trajectories in the state space by tracking consecutive sequences of points. First, for each vigilance-state the state space densities were plotted and all values up to 20% of the maximum value were included in a boundary for each vigilance-state. Trajectories connecting different clusters were considered to be valid transitions if: 1) the duration of the trajectory connecting two different clusters was 60 seconds and 2) the trajectory spent at least half of the preceding 30 seconds in the initiating cluster and half of the 30 subsequent seconds in the terminating cluster. In addition, we explored the effect of UAO on microarousal during the first 6 hours of lights on, a period where animals’ sleep is maximal [21]. Microarousal event was defined as a short trajectory event to wake state that lasted ≥3 and up to 15 seconds.

### Data Analysis

Sleep/wake dynamics and statistical analyses were performed using MATLAB (R-2008b, The MathWorks, Inc., Natick, MA, USA). Significance was analyzed by student *t*-test or Wilcoxon Rank-sum test as appropriate. Two-way analysis of variance for one-repeated measure was used to determine significance between time and groups or time and drug using post hoc comparisons by Student–Newman–Keuls test. Null hypotheses were rejected at the 5% level.

## Results

A total of 19 animals were included in this study; n = 10 and n = 9 for the UAO and control groups, respectively. UAO rats’ behavior was similar to that of the controls; they explored their cage and engaged in social activity such as grooming. All the animals demonstrated audible wheezing, especially after activity, but no signs of respiratory distress or gasping at rest were observed among UAO animals. As expected, following tracheal obstruction surgery, there were signs of increased mechanical resistive loading indicating that the trachea was mildly to moderately obstructed [9], [10], [13], [16], [20], i.e., inspiratory swings in esophageal pressure (ΔPes) increased from −3–5 (cm H_2_O) to −14–19 (cm H_2_O) (*p* = 0.03) and respiratory rate decreased from 91±15 (breaths/min) to 70±19 (breaths/min) (*p*<0.001).

### Vigilance-states

Traditional scoring of vigilance-states indicated that both groups exhibited circadian rhythms of wake, SWS, and PS. There were, however, several significant differences in sleep between UAO and control groups (Figure 1). The UAO group was 43.9% more awake during the light period (*p*<0.01, ANOVA-2) and had 19.7% less SWS duration during lights-on period (*p*<0.01, ANOVA-2). UAO animals had considerably less PS (*p*<0.01 ANOVA-2) than controls during both light (47.2%, *p*<0.001, ANOVA-2) and dark periods (34.5%, *p*<0.01, ANOVA-2) (Figure 1).

### State Space Technique

Both groups’ vigilance-states of wake, SWS, and PS were consistently mapped to distinct regions of 2D state space and the clusters correspond to the conventional scoring of these states. The dispersion of points within each cluster reflected variations in sleep depth or its intensity of wakefulness, as measured by EEG magnitude in the delta and theta bands, respectively. Figure 2A,B shows raw scatter plot from one control and one UAO animal. Each plot shows 24 hours of EEG activity, and each point represents 1 second of EEG activity. These 2D plots were further smoothed with a Hanning window (20-second length) (Figure 2C,D) and enable identification of behavioral states or transition between behavioral states (Figure 2E,F). The SWS cluster was always located on the right middle center of the 2D state space plot, whereas PS and wake clusters occupied center and left regions, respectively. The Wake cluster is located at low ratio 1 values, while SWS is predominantly located at high ratio 1 values. PS clusters could be found between wake and SWS clusters and they were almost missed in the UAO group. SWS cluster was reduced, while wake cluster shifted to lower ratio 1 value in UAO rats. Furthermore, UAO led to considerable change in SWS composition. Color separation of LSWS and DSWS revealed that UAO animals have considerably more LSWS and reduction of DSWS (Figure 2E,F). We found no evidence for significant changes in ratio 2 values (y axis) between groups (Figure 2E,F).

**Figure 3:**
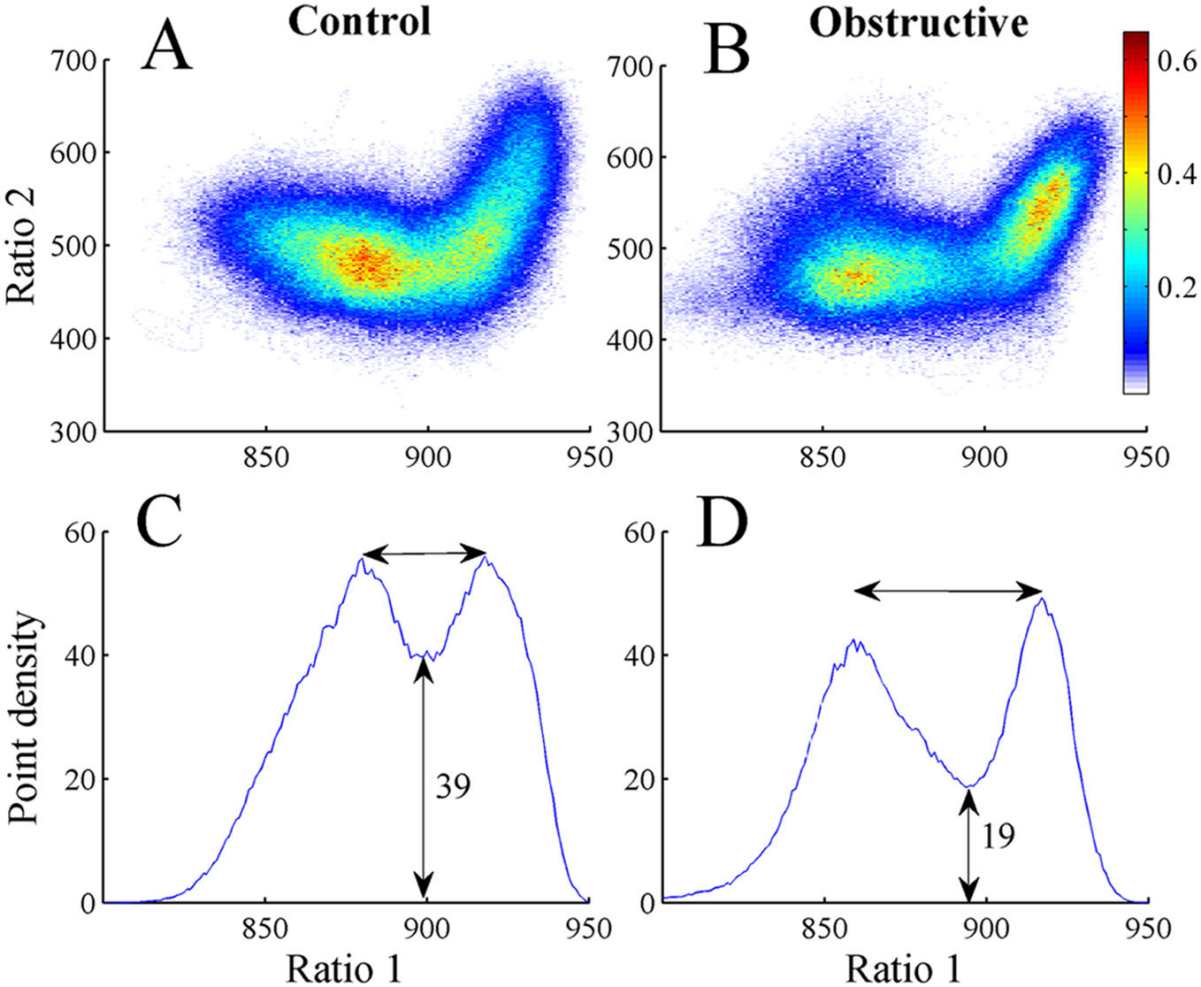
Point densities averaged across all control (A) and UAO (B) animals. Warm colors indicate regions where the average density is high and cool colors indicate low density. (C), (D)–average state space densities for control and UAO rats projected into ratio 1. This projection yields two peaks; left peak is associated with wake and the right peak is associated with SWS sleep.

**Figure 4:**
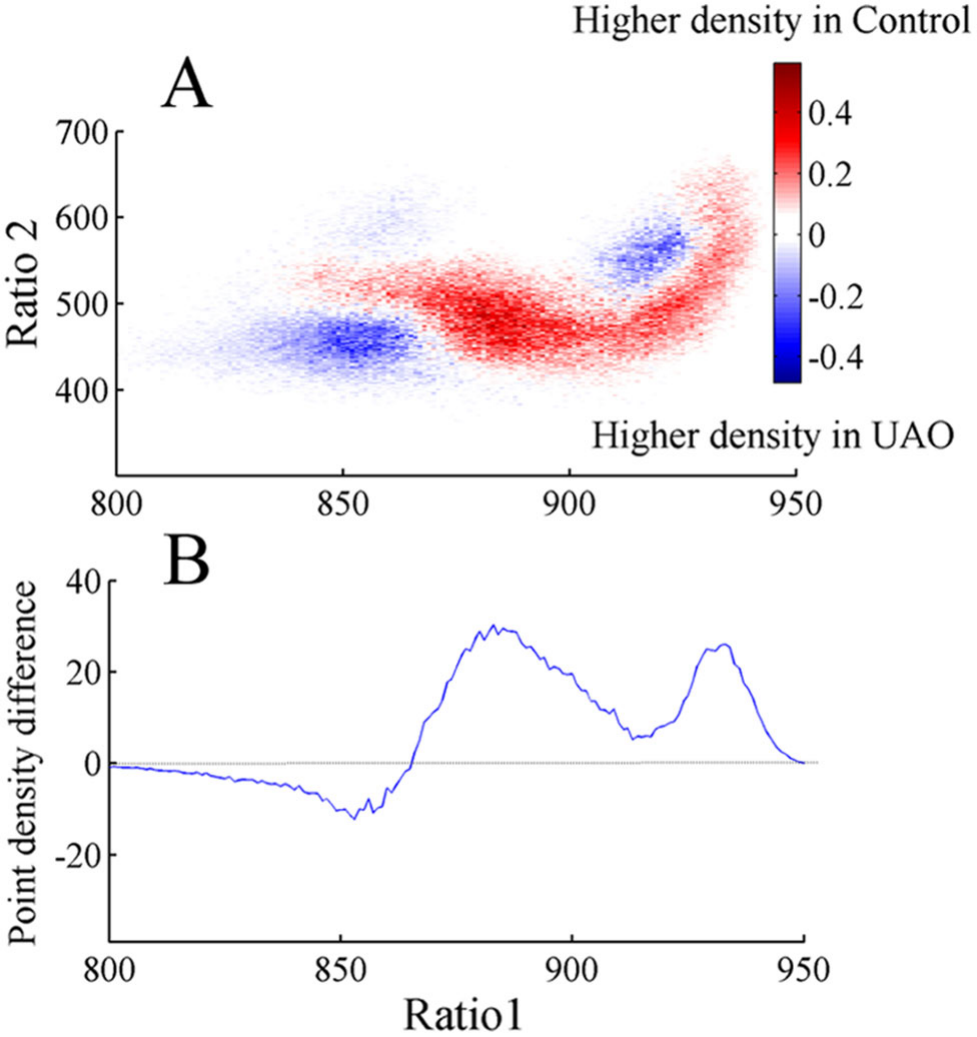
Difference plot showing average density pattern of UAO rats subtracted from control rats (A). B–projection of this graph into ratio 1. The color scale highlights differences between the groups: warm colors indicate regions where the average density is higher in controls and cool colors indicate higher density in UAO group.

Point densities of the 2D state space plots were averaged in control and UAO groups (Figure 3A,B). Warm colors indicate regions where the average density is high and cool colors specify low density. Figure 3 reveals two peaks in both control and UAO animals corresponding to wake (low ratio 1) and SWS (high ratio 1) states. To further quantify these peaks, mean point densities were projected into ratio 1 for control (Figure 3C) and UAO (Figure 3D) animals. The point density in the region between clusters is reflected by the height of the lowest point between peaks (vertical arrow, Figure 3C,D). The mean lowest point between peaks in the control and UAO rats was significantly lower in UAO rats, 39±1.4 (arbitrary units) and 19±0.5 (arbitrary units) (*p*<0.05), respectively. This finding indicates that UAO group spent less time in the transition region between wake and SWS. We also calculated the horizontal distance between peaks. The peak-to-peak horizontal distance tended to increase by 36% in UAO animals (*p* = 0.06) due to movement of the left peak of ratio 1 (corresponding to wake) from 890 to 850 in the control and UAO groups respectively; while the right peak of ratio 1 did not change (Figure 3C,D). This finding indicates that UAO leads to increased wake frequency and intensity. To further understand the effect of UAO on vigilance-states, the average density plot of the UAO group was subtracted from the average control group density plot (Figure 4A); we plotted the projection of this graph to ratio 1 (Figure 4B). The UAO group has increased wake cluster frequency, indicated by the blue region in the left side of Figure 4A (or by the negative values in Figure 4B) and less DSWS as specified by the red region in the right side of (Figure 4A). To ensure that the inclusion of PS did not affect our findings, we eliminated data scored as PS sleep and reanalyzed the data. The results of that analysis were very similar (data not shown).

### State Space Velocities

**Figure 5:**
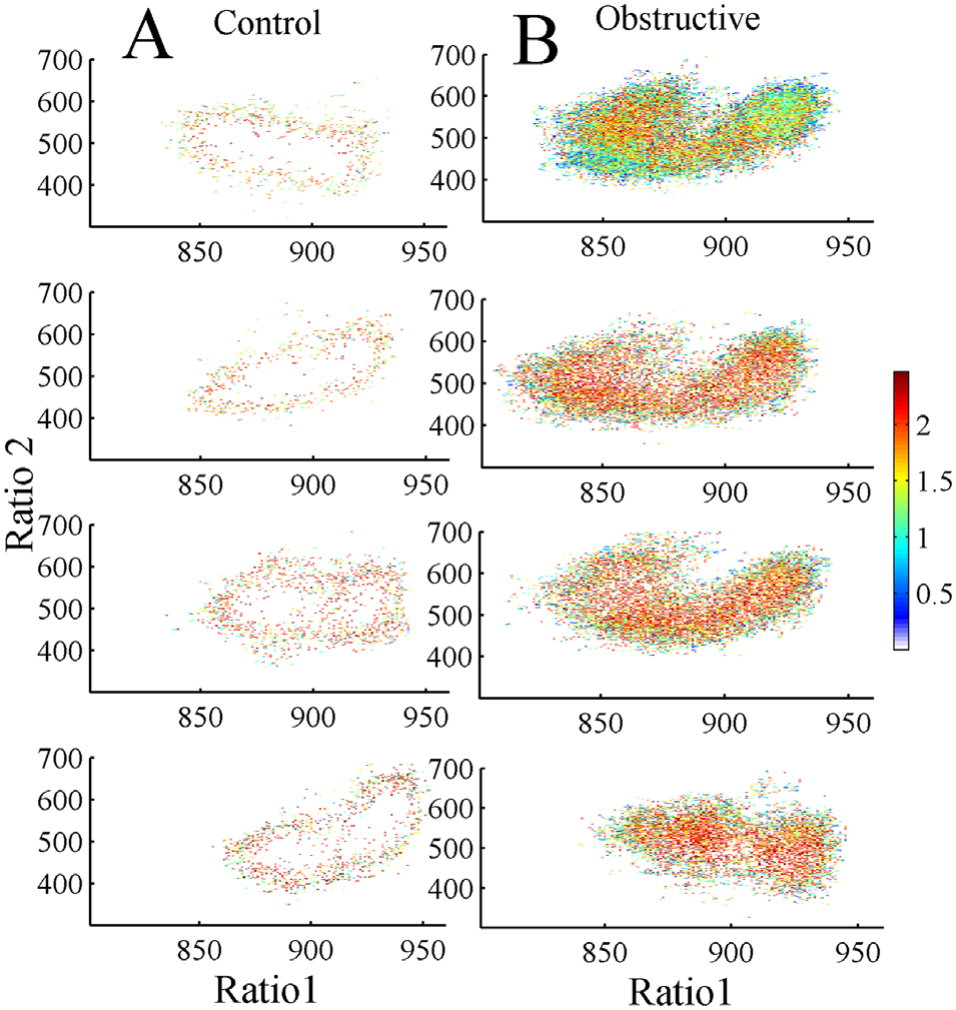
UAO group has faster movements in all regions of the 2D state space plot compared to controls. Plots represent four different control (A) and UAO (B) animals. The velocity of spectral change was calculated as the distance between two consecutive data points and so temporal resolution was second-by-second. Values represent average velocities originating at that site. Warm colors show faster velocity (transition regions) indicating unstable states, and cool colors low velocities at stable states. UAO animals have faster movements.

**Figure 6:**
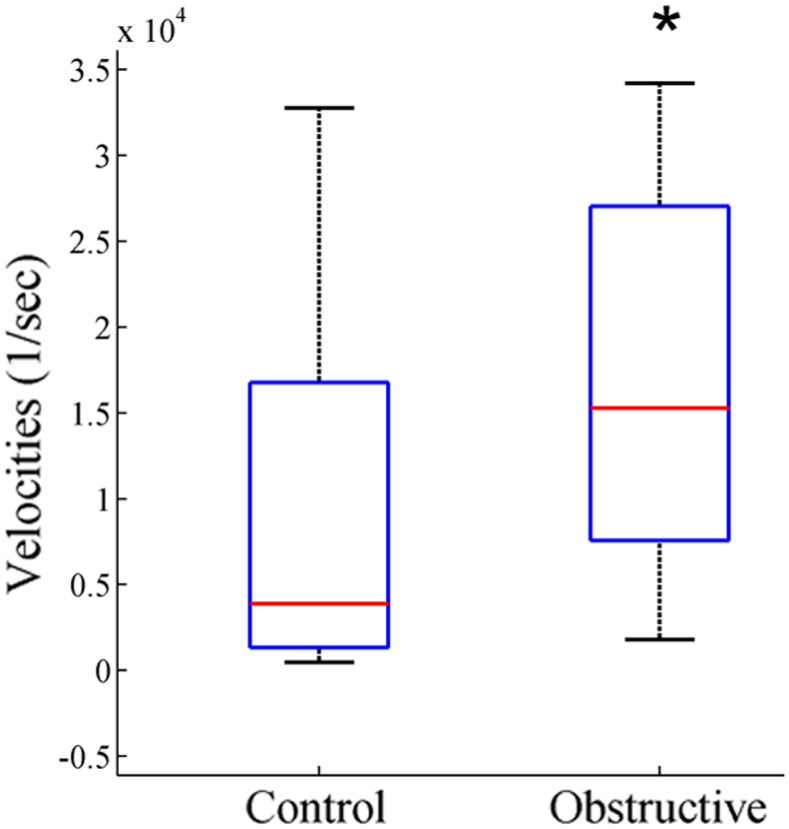
Box plot shows the median velocity of control and UAO groups following calculation of the sum of the velocities in each animal. *p<0.05.

Figure 5 illustrates an example of individual velocity plots for 4 control (Figure 5A) and 4 UAO (Figure 5B) animals. A value at each point represents average velocities originating at this site. The velocity of the UAO group was much faster than that of controls in all regions of the 2D state space plot. This finding indicates that the UAO group has more regions where sleep/wake states are less stable. The median velocity (Figure 6) of the UAO group increased by 450% compared to the control group (*p* = 0.03).

### Trajectories between Vigilance-states

**Figure 7:**
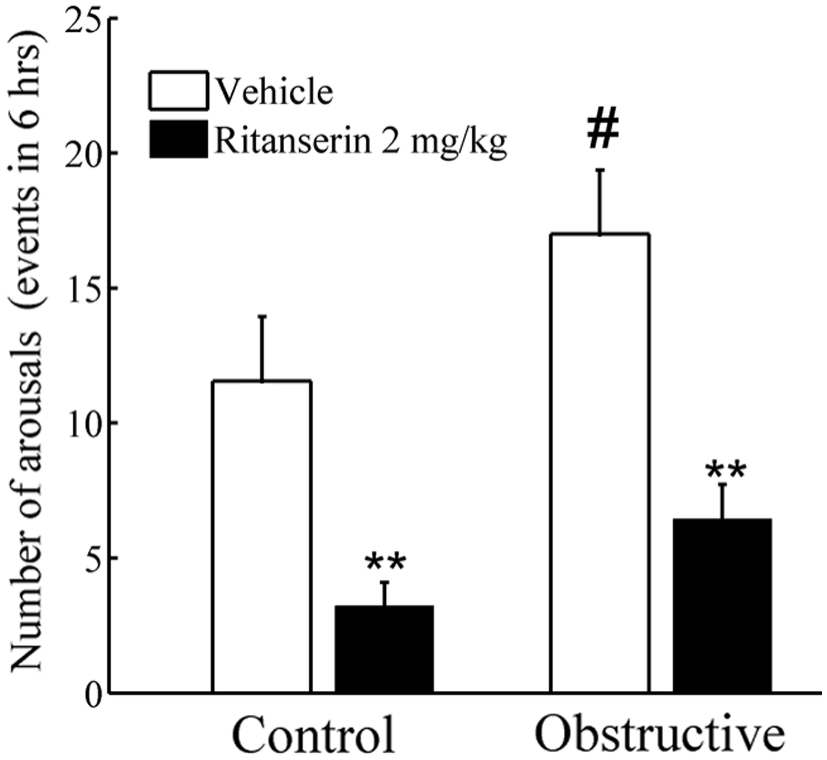
Effect of ritanserin on microarousal during the first 6 hours of lights on. UAO group has significantly more microarousals. Treatment with ritanserin reduces the number of microarousals. **p<0.001– comparing baseline vehicle to ritanserin. #*p*<0.05– comparing controls to UAO group.

To explore the patterns of 2D state space dynamics as rats move between vigilance-states, we identified trajectories in the 2D state space by tracking consecutive sequences of points. Using a contour algorithm (see Methods), we delineated cluster core boundaries for each vigilance-state (Table 1). For all vigilance-states, all possible moving combinations of trajectories were calculated. Common trajectories were from LSWS to DSWS and vice versa, and from wake to LSWS and vice versa. These trajectories accounted for 55% and 76% of all trajectories in the control and UAO animals, respectively (Table 1). The numbers of trajectories from wake to LSWS (*p* = 0.004) and vice versa (*p* = 0.02) were 312% and 268% higher in the UAO group, respectively. Trajectories from PS to LSWS (*p* = 0.03) and DSWS to PS (*p* = 0.008) were considerably lower in the UAO group. Brief vigilance-state microarousal changes from sleep to wake of >3 and up to 15 seconds were calculated. The number of microarousals during the first 6 hours of lights on, a period of maximal sleep (Figure 1), was 45% higher in the UAO group compared to controls (*p* = 0.05, Figure 7). In both groups about 80% of these microarousal events occurred during LSWS.

**Table 1:** Mean number of trajectories between vigilance-states.

Vigilance State		Control(mean, %)	Obstructive(mean, %)	*p* value
Total number of trajectories		149.5±29.4 (100%)	182.7±19 (100%)	0.24
From stage	To stage			
LSWS	DSWS	35.3±4.2 (22.3%)	33.5±5.2 (17.7%)	0.89
DSWS	LSWS	21.8±2.6 (13.7%)	19.8±3 (10.5%)	0.81
Wake	LSWS	17.7±1.7 (11.1%)	55.2±9.7 (29.1%)	0.004
LSWS	Wake	13.7±1.3 (8.6%)	36.8±5 (19.4%)	0.002
LSWS	PS	12.8±1.2 (8.1%)	5.2±2 (2.7%)	0.08
PS	Wake	12.1±1.2 (7.6%)	11.5±3 (6.1%)	0.89
Wake	DSWS	9.3±1.3 (5.9%)	3.3±1.1 (1.7%)	0.15
PS	LSWS	8.8±0.9 (5.5%)	2.0±1.5 (1.1%)	0.03
DSWS	Wake	8.1±1.2 (5.1%)	5.3±1 (2.8%)	0.44
Wake	PS	6.1±1.1 (3.9%)	9.8±2.4 (5.2%)	0.38
DSWS	PS	3.0±0.3 (1.9%)	0.3±0.2 (0.2%)	0.008
PS	DSWS	1.2±0.1 (0.8%)	0.0	–

Data show the number of spontaneous transitions between behavioral states collected from 456 hours of a rat’s life. W–Wake; LSWS–light slow wave sleep; DSWS–deep slow wave sleep; PS–paradoxical sleep. Values are mean±SEM. (%) Percent of total number of trajectories.

### Effect of Ritanserin on Sleep

**Figure 8:**
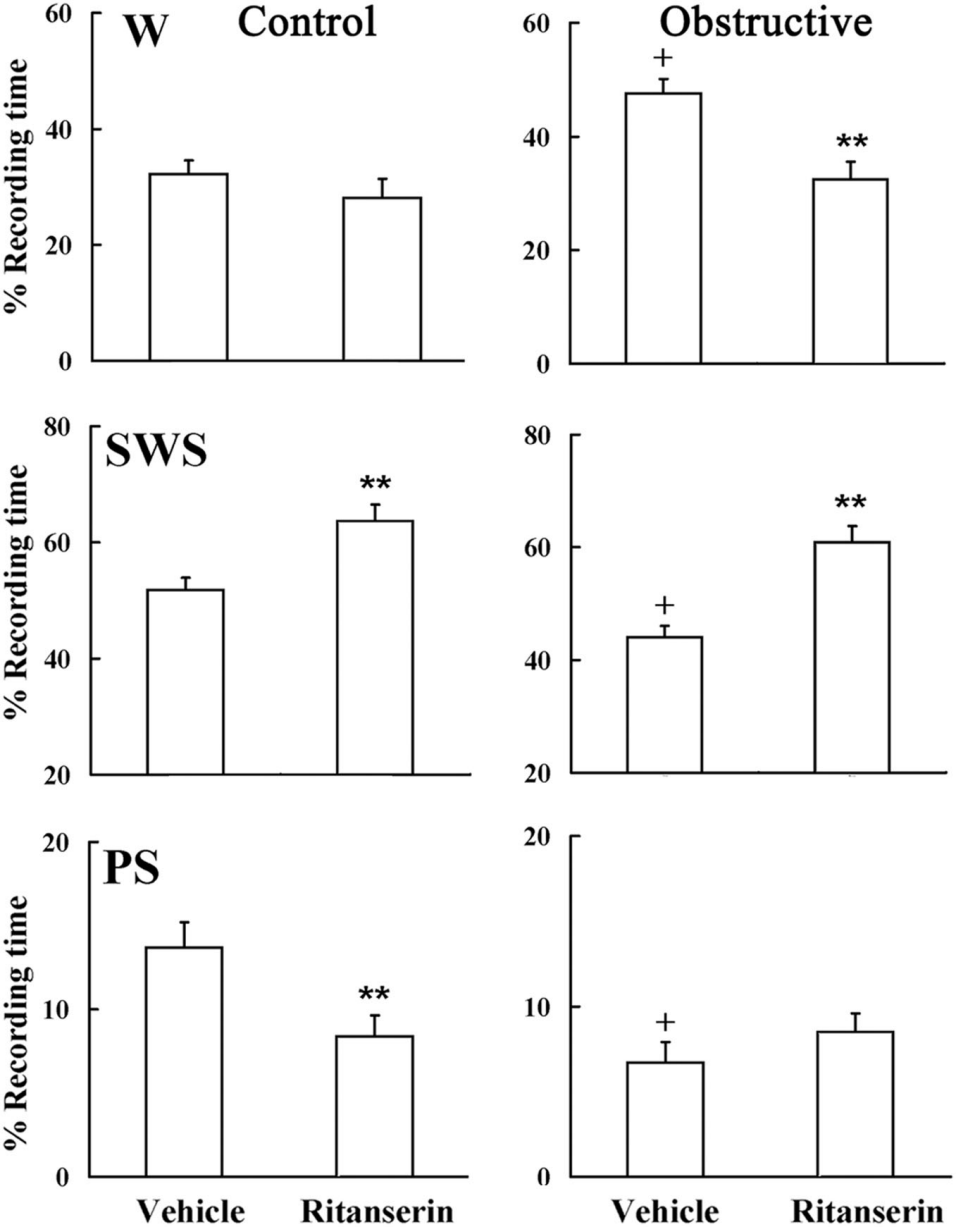
Effect of ritanserin on spontaneous sleep during first six hours of light-on period. On day one animals were given vehicle (4% methyl alcohol in saline) and on day two animals were treated with ritanserin (2 mg/kg) at lights on. W–wake, SWS–slow-wave sleep, PS–paradoxical sleep. **p<0.001– comparing vehicle to ritanserin study; + *p*<0.001 comparing vehicle study of control rats to obstructive rats. Values are mean (SEM).

**Figure 9:**
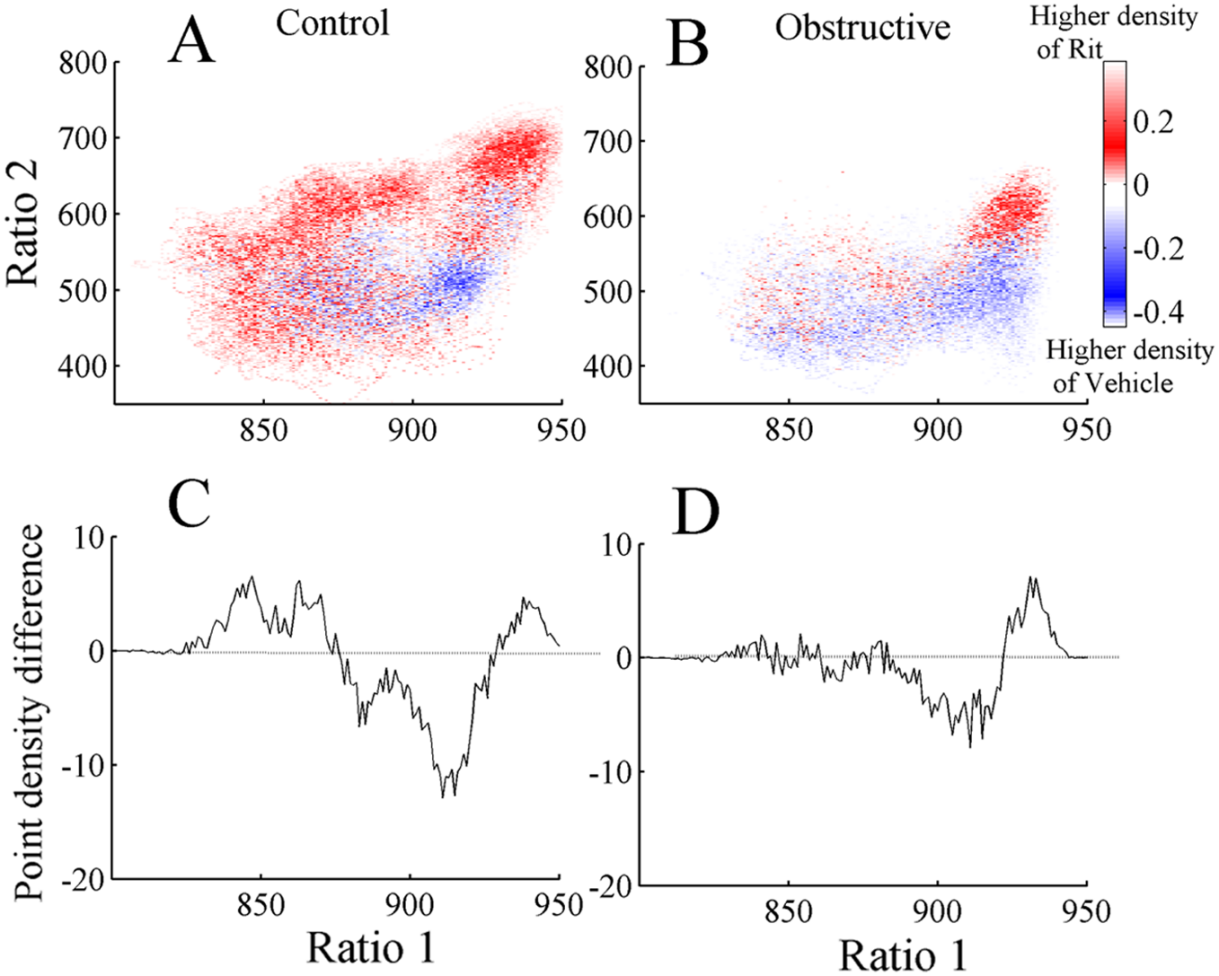
Mean difference averaged densities during first 6 hours lights on in control (A) and UAO (B) groups. Differences were calculated by subtraction of baseline vehicle study from the ritanserin (2 mg/kg) study. Warm colors indicate regions where the average density of the ritanserin study is higher relative to vehicle study and cool colors indicate higher density of vehicle study. In both groups ritanserin increased delta rich deep SWS and decreased light SWS. Projection of ratio 1 (C) control and (D) UAO group.

Conventional scoring of sleep (Figure 8) shows that during baseline vehicle, UAO rats had 42% more wake (*p*<0.001), 17% less SWS (*p*<0.001), and 56% less PS (*p*<0.001) (Figure 8). Acute administration of high dose 5-HT2 receptor antagonist did not significantly affect wake duration in the control group compared to baseline vehicle recording (Figure 8). In the UAO group, ritanserin significantly decreased wake duration during by 31.7% (*p*<0.001) during the first six hours of lights on in the UAO group compared to baseline vehicle recording (Figure 8). Ritanserin significantly increased SWS by 20.5% (*p* = 0.01) and 38.4% (*p*<0.001) during the first six 6 hrs of lights on in the control and UAO groups, respectively. Treatment with ritanserin significantly decreased PS in the control group by 38% (*p*<0.01). In both groups ritanserin increased delta-rich deep SWS and decreased light SWS. Figure 9A,B shows the subtraction of 2D state space frequency ratios of the baseline vehicle from the ritanserin study during the first 6 hours of lights on in control and UAO groups, respectively. Warm colors indicate regions where the average density of the ritanserin study is higher relative to vehicle study and cool colors indicate higher density of vehicle study. Figure 9C,D shows the projections (see methods) of Figure 9A,B to ratio 1; positive values indicate higher density of ritanserin relative to vehicle and negative values indicate vice versa. In both groups ritanserin increased DSWS while LSWS decreased at the same time. However, ritanserin did not affect the velocity of the state space plot (data are not shown).

**Figure 10:**
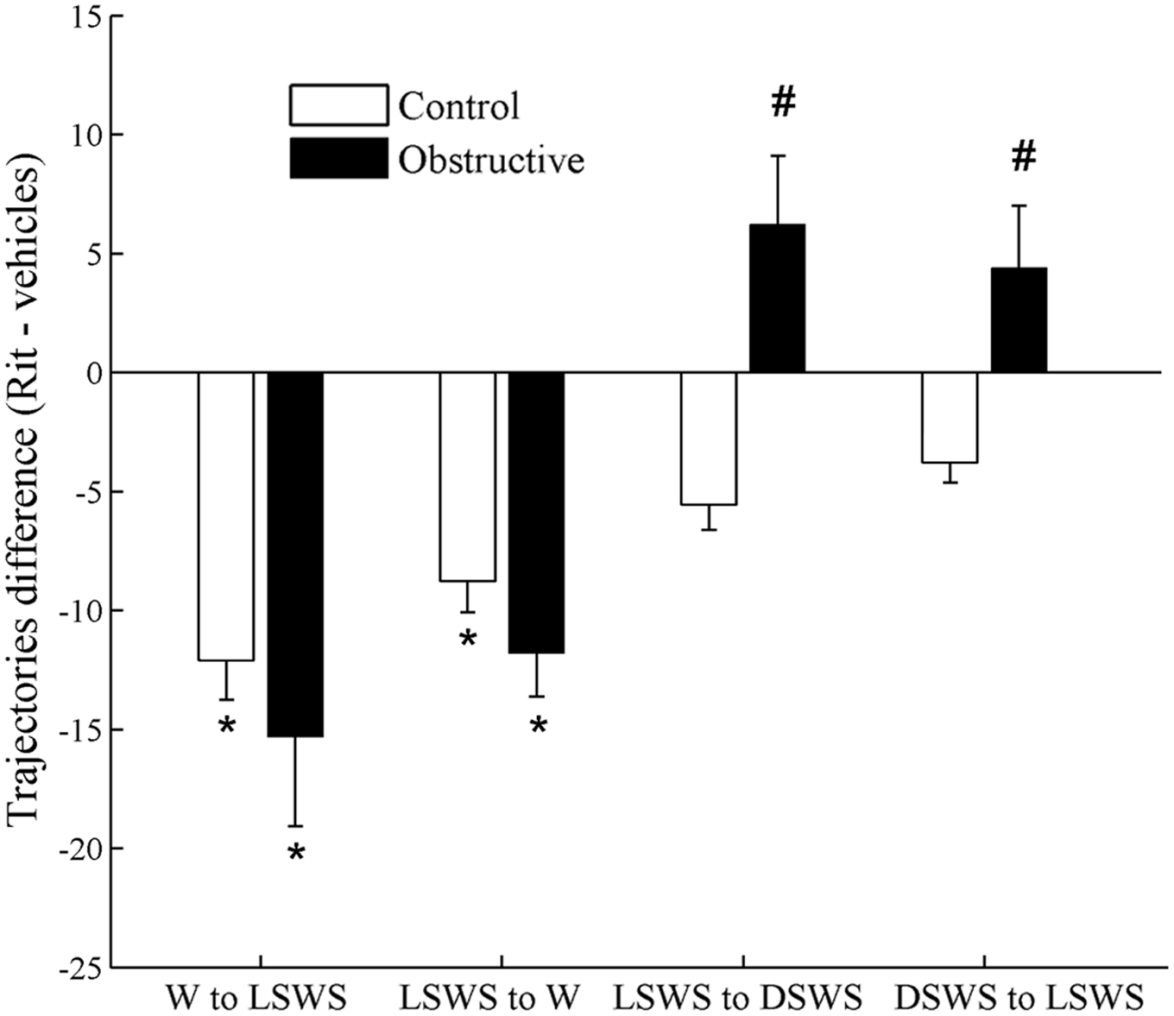
Effects of ritanserin on differences in number of trajectories. Difference in number of trajectories was calculated by subtraction of ritanserin results from baseline vehicle measurements. In both groups the number of trajectories significantly decreases between wake and light SWS (LSWS) and vice versa after ritanserin (Rit) administration. Ritanserin significantly increased the number of trajectories of LSWS to deep SWS (DSWS) in UAO group. *p<0.05 comparing ritanserin results to baseline vehicle measurements in the same animals. #*p*<0.05 comparing change in trajectories between control and UAO groups.

Figure 10 shows the difference in the number of trajectories following treatment with ritanserin. In both groups we found similar decreases in the number of trajectories from wake to LSWS and vice versa (*p*<0.05). Ritanserin significantly increased the number of trajectories from LSWS to DSWS and vice versa in the UAO group (*p*<0.05), but not in the control group. Ritanserin decreased the number of microarousal events by 72% (*p*<0.001) and 62% (*p*<0.001) in control and UAO groups, respectively (Figure 7).

## Discussion

Here, we present evidence that UAO induces substantial temporal alterations in sleep-wake activity, indicating fragmented sleep, which was not fully apparent when analyzing sleep by conventional methods. UAO animals spent less time in typically “stable” areas of wake and SWS. UAO induced faster velocities between all vigilance-states, more trajectories from wake to LSWS and vice versa, and higher microarousal events during the first 6 hours of lights on. Administration of ritanserin consolidated sleep by decreasing the number of trajectories from LSWS to wake, decreasing the number of microarousal events, and increasing DSWS in the UAO group.

### UAO Model Strength and Limitation

To our knowledge this is the first study exploring the effects of UAO sleep dynamics in juvenile rats. UAO was induced in 22-day-old rats, and animals were followed for 16 days, a period that is comparable to the range of six months to eight years of age in children [15], [16]. This animal model’s strength and limitation was previously discussed in several studies [9], [12]–[16], [20]. Briefly, the reduced respiratory rate and inspiratory swings in esophageal pressure in the current study indicate that the trachea was mildly to moderately obstructed, and these effects were not exclusively sleep related. In this model both inspiratory and expiratory loading were induced without evidence for obstructive apnea or hypopnea [13]. This condition may resemble tracheal stenosis [30] or upper airway resistance syndrome that are associated with large swings in intra-thoracic pressure and sleep fragmentation [16], [20], even in the absence of frank apneas/hypopneas or gas exchange abnormalities [31]. Sleep-disordered breathing is associated with intermittent upper airway obstruction at night, primarily during inspiration and is sleep related [32]. Under these conditions animals were able to maintain ventilation and arterial PO_2_ [13]–[16], [20]. It seems likely that our model also has implications for this condition since UAO animals have abnormal sleep [16], [20] similar to sleep-disordered breathing as seen in the subset of children with this disorder [1], [3].

### Behavioral State Instability

We used the SST to explore vigilance-state dynamics in chronic UAO juvenile rats. This approach provides a novel, non-categorical method for analyzing sleep/wake behavior in chronic UAO rats. The 2D SST has a high temporal resolution and analyzes behavior as a continuum, rather than discrete states, and so facilitates higher dimensional examination of state transitions [21]–[24]. Traditional spectral analysis often excluded or diluted events through averaging. In addition, this approach employs the ratio of two frequency bands rather than a single frequency band. We determined whether UAO animals have state instability reflecting abnormal sleep/wake states, faster movements between states, abnormal transition processes, and fragmented sleep. The general location of state space clusters SWS and PS are conserved in UAO rats [16], [20]. However, we identified several differences between groups. The density graph analysis indicates that the SWS cluster did not change between control and UAO, while the wake cluster shifted to lower ratio 1 in the UAO group (Figure 3C,D). The UAO group has a higher velocity at all regions of the 2D state space plot, suggesting less stable vigilance-states. UAO leads to more trajectories between wake and LSWS and vice versa and higher microarousal index, indicating that obstructed animals have fragmented sleep. In children it was reported that early adenotonsillectomy leads to significantly larger decrease in the arousal index and in the percentage of sleep time in stage N1 (light sleep), consistent with improved sleep continuity [2]. Transitions between wake and SWS in UAO rats do not originate in extreme regions of the deep (delta-rich) SWS (highest ratio 1) and PS. UAO animals spent less time in typically “stable” areas of wake and SWS. This reduction in delta-rich SWS in UAO is consistent with the reduction in slow-wave activity in rats [16], [20] and in humans with sleep-disordered breathing [7], [33]. Administration of ritanserin has a strong sleep consolidation effect in both groups, similar to earlier reports in rats [26] and in humans with preexisting sleep fragmentation [34]. Similar to other reports [27], [35], the effects of ritanserin on sleep/wake pattern are limited to the first hours of light onset following drug administration due to its known pharmacokinetics. The improvement of sleep-wake activity in our study following ritanserin was due to increased time spent in stable regions of the DSWS cluster, less fragmented sleep, and decreased number of microarousals from LSWS. Ritanserin, which has a role in regulating SWS depth, stimulates hypothalamic growth-hormone-releasing hormone secretion in UAO [16], [29], [36], [37]. It is possible that up regulation of hypothalamic orexin in UAO rats [20] has an important role in this sleep fragmentation [19], [38]–[40].

SST of the EEG is especially useful for studying the dynamics of sleep/wake instability, but it has some limitations. Comparison of trajectories and microarousals after different EEG recording times could be problematic since they are calculated according to the state boundaries. The clusters determine the boundaries of the distinct states and so higher cluster densities due to longer EEG recording increased the size of the boundary. Also, the separation between the wake and PS clusters was less well defined using the surface EEG signal than it was with depth recordings, probably due to better detection of hippocampal theta activity with depth electrodes [21]. Previous work has shown a high degree of coherent activity in cortex, hippocampus, striatum, and thalamus across states in normal animals [21] and humans [41], suggesting that a single channel of EEG is sufficient for state space analysis of sleep/wake behavior. The electrode locations in our study did not permit assessment of coherence. Future investigations with a more detailed EEG montage could use state space analysis to examine whether reduced cortical coherence contributes to sleep/wake instability in UAO rats.

In pediatric sleep-disordered breathing, severity of the disorder is assessed by polysomnography. Although standard polysomnography measures are based on recorded rates of pathological respiratory events and at least in part through sleep fragmentation, previous comparison polysomnography data have failed to explain important outcomes, such as excessive daytime sleepiness and neurocognitive abnormalities [42]–[45]. Studies exploring distributions of contiguous sleep duration following adenotonsillectomy also found no consistent polysomnography findings [2], [8], [46]–[48]. The notion was that children with sleep-disordered breathing do not rouse from their respiratory events during sleep as often as adults do; therefore, sleep architecture is better preserved than in adults [7], [8], [47]. The cumulative data on the effect of adenotonsillectomy on sleep stages using traditional polysomnography scoring are probably too small to carry the obvious consistent improvement in clinical, neurocognitive, growth, and endocrine changes following adenotonsillectomy in children with sleep disordered breathing. As increasingly sophisticated genetic and physiologic techniques are applied to probe neuronal mechanisms involved in sleep/wake regulation, new measures of sleep/wake behavior are needed [49]. Efforts to improve assessment of brain activity during sleep have focused on arousals [50], alternative EEG leads to detect them [51], respiratory event-related arousals [52], and EEG signal analysis; or changes in EEG spectral power before and during obstructive events during sleep [53]. However, most of these approaches remain inextricably linked to apneas or other discrete, visually identified respiratory events, the rates of which are known to be sub-optimal correlates of health outcomes. It is possible that by enabling visualization of behavioral states as a continuum, SST captures the richness of physiology better than conventional, categorical scoring of sleep/wake behavior. The fine temporal resolution of SST allows investigation of state transitions and transition dynamics that is not possible with traditional methods. Further studies should determine the temporal link state-space dynamics with respiratory instability in UAO rats using inspiratory swings in pleural pressure and sleep recording [54], and the usefulness of SST in addition to standard sleep stage analyses in children with sleep-disordered breathing.
